# Point-of-care lung ultrasound for diagnosis of *Pneumocystis jirovecii* pneumonia: notes from the field

**DOI:** 10.1186/s13089-018-0089-0

**Published:** 2018-04-17

**Authors:** Maria Teresa Giordani, Francesca Tamarozzi, Daniel Kaminstein, Enrico Brunetti, Tom Heller

**Affiliations:** 1 Infectious and Tropical Diseases Unit, San Bortolo Hospital, Via Rodolfi 37, 36100 Vicenza, Italy; 2 Center for Tropical Diseases, Sacro Cuore-Don Calabria Hospital, Negrar, Verona Italy; 3 Department of Emergency Medicine and Hospitalist Service, Medical College of Georgia at Augusta University, Augusta, GA USA; 4 Division of Infectious and Tropical Diseases, University of Pavia, IRCCS S. Matteo Hospital Foundation, Pavia, Italy; 5 Lighthouse Clinic, Lilongwe, Malawi

**Keywords:** POC ultrasound, Lung, *Pneumocystis* pneumonia, AIDS, HIV, Pneumonia, Lung ultrasound

## Abstract

**Background:**

Thoracic ultrasound is helpful to evaluate lung pathology in patients with acute dyspnea. Several studies have demonstrated the efficacy of point-of-care ultrasound in patients with extrapulmonary TB and HIV co-infection. This retrospective, open-label case–control study explores the role of lung ultrasound in the diagnosis of *Pneumocystis jirovecii* pneumonia (PJP) in HIV-positive patients. In particular, it highlights the potential role of specific sonographic features that may be unique to this population.

**Methods:**

The record of all HIV-positive patients admitted from 1.1.2013 to 31.6.2017 to the Department of Infectious Diseases and Tropical Medicine of san Bortolo Hospital, Vicenza, Italy, with a discharge diagnosis of acute lung injury (ALI) and who received point-of-care ultrasound of the chest for clinical purposes was included in the analysis. The patients were scanned according with the evidence-based recommendation.

**Results:**

Of 273 HIV-positive patients whose records were reviewed, 81 (29.6%) were diagnosed with ALI. Complete documentation was available for 24 patients, of which 14 (58.3%) had microbiologically confirmed PJP (PJP+) and 10 (41.7%) had other conditions (PJP−). B-lines, subpleural consolidations, and cystic changes were significantly more frequent in patients with PJP (14/14 vs. 6/10, *p* = 0.0198; 14/14 vs. 4/10, *p* = 0.0016; 8/14 vs. 0/10, *p* = 0.0019, respectively). In particular, B-lines and subpleural consolidations were present in all PJP+ patients in our cohort giving a sensitivity of 100%, but their specificity was low (45 and 60%, respectively). On the contrary, the presence of consolidations with cystic changes had a very high specificity for PJP (100%), but low sensitivity (57%). Pleural effusions and consolidations with linear air bronchograms were not observed in PJP+ patients.

**Conclusions:**

B-lines, subpleural consolidations, and cystic changes are suggestive of PJP. Lung consolidation with air bronchograms and pleural effusion should prompt suspicion of other etiologies. These findings have the potential to be useful in the daily management of HIV-positive patients in resource-limited settings where other diagnostic tools are rarely available.

**Electronic supplementary material:**

The online version of this article (10.1186/s13089-018-0089-0) contains supplementary material, which is available to authorized users.

## Background

The interpretation of artifacts created during ultrasound of the thoracic cavity was first described over 20 years ago [1]. Since then thoracic ultrasound has become a well-established imaging modality used by clinicians at the bedside of acutely dyspneic patient. The BLUE protocol [2] is a helpful approach to lung ultrasound as it outlines a step-by-step decision tree to differentiate between potential causes of dyspnea. In low-resource settings where even a basic chest X-ray (CXR) may not be available [3] ultrasound of the chest has profound implications. Point-of-care ultrasound (POCUS) has an established role as a diagnostic tool in HIV-positive patients with extrapulmonary tuberculosis (TB) [4], and has been shown to complement CXR [5]. Furthermore, it is a frequent POCUS application in settings with high TB and HIV prevalence [6]. Recent case series describe sonographic lung finding seen in HIV-positive patients [7] and in patients with miliary TB [8].

*Pneumocystis jirovecii* pneumonia (PJP) is one of the most common opportunistic infections in HIV-positive patients [9] who are not receiving prophylaxis especially when CD4− counts fall below 200 cells/mm^3^ [10]. Without timely diagnosis and treatment PJP carries a high mortality [11], though survival rates have increased in the era of antiretroviral therapy (ART) [12]. Diagnosis of PJP poses a significant challenge, due to its non-specific clinical presentation. Computed tomography (CT) of the chest remains the mainstay of diagnosis [13]. In cross-sectional CT imaging, PJP presents with bilateral geographic or patchy ground-glass opacities with septal thickening, lung consolidation, nodules, cysts, and pneumothorax, while pleural effusions are uncommon [14]. Microbiological confirmation relies on bronchoalveolar lavage (BAL) followed by microscopy or PCR. BAL is invasive, poorly tolerated by patients with respiratory distress and analysis requires a well-equipped laboratory with specific expertise.

In resource-limited settings, where the majority of HIV-positive patients live, the risk of PJP is high [15]; CT and bronchoscopy are generally not available. As POCUS has been found a promising tool for other diseases in these settings [16, 17], our study aimed to explore the diagnostic potential of sonographic lung features in the diagnosis of HIV-positive patients with PJP.

## Methods

### Study design and data collection

This retrospective unmatched case–control study of HIV-positive patients with and without microbiologically confirmed PJP was conducted in the Tropical Medicine and Infectious Diseases Department, San Bortolo Hospital, Vicenza, Italy, that cares for approximately 1000 HIV-positive individuals yearly.

The department operates a clinical ultrasound service including bedside POCUS. Chest POCUS is performed by an infectious disease specialist (MTG) using an Aplio XG Model SSA-790A with a 3.5-MHz convex and an 8-MHz linear probe (Toshiba, Tokyo, Japan). All patients included in this study were examined with both probes; the anterior, posterior, and lateral chest wall was bilaterally scanned in longitudinal (cranio-caudal orientation) and transverse (aligned with the intercostal space) orientation from apex to base with patients in supine, sitting or near-to-supine position, trying to achieve the maximum extension of the visible pleural line. No standard protocol was followed, although the International evidence-based recommendations for point-of-care lung ultrasound were taken into consideration [18].

All records of HIV-positive patients admitted from 1.1.2013 to 31.6.2017 were reviewed and patients with a discharge diagnosis of acute lung injury (ALI) according to Diagnosis-Related-Group (DRG) classification (http://www.salute.gov.it/imgs/C.pdf) were identified. Inclusion criteria were as follows: (1) HIV infection; (2) discharge diagnosis of ALI; (3) BAL followed by microbiological examination for *P. jirovecii* (microscopy or PCR); and (4) POCUS of the chest performed during the first week of admission blinded to CT scan findings. Extracted patient information included demographics, history of ART, CD4+ count, HIV viral load, microbiological, and imaging data. Stored static images and video files of all patients were reviewed for the study (Additional files 1, 2, 3).

### Statistical methods

Patients included were classified as microbiologically confirmed PJP (PJP+) or control patients with ALI without microbiologically confirmed PJP (PJP−). Data are presented as medians, interquartile ranges (IQR), and counts with percentages, as appropriate. Differences in proportions were analyzed by Fisher’s Exact test; continuous variables by Mann–Whitney-*U* test. All tests were two-sided; *p*-values < 0.05 were considered significant. Analyses were done using MedCalc version 17.8 (Ostend, Belgium).

### Description of ultrasound findings

An ultrasound was considered normal when a normal lung surface, a pleural line with lung sliding, and A-lines were present and a maximum of two B-lines per single view was seen. Pathological ultrasound findings extracted for analysis were increased number of B-lines, pleural effusions, subpleural consolidation, lung consolidation with air bronchogram patterns or a cystic pattern, and presence of pneumothorax. Ultrasound findings are described in detail in Table 1.

**Table 1:** Clinical and demographic characteristics of the patients included in the study

	Total *n* = 24 (100%)	PJP+ *n* = 14 (58.33%)	PJP− *n* = 10 (41.66%)	*p*-value
Sex males/females (% males)	18/6 (75.00%)	13/1 (92.85%)	5/5 (50.00%)	0.0501
Median age in years (IQR)	44 (36–53)	43 (35–54)	44 (41–51)	0.8602
Ethnic origin (*n*)				
Caucasian	18	11	7	
African	3	1	2	
Latin-American	3	2	1	
Median CD4+ T cells/μl on admission (IQR)	56.5 (18–172.25)	39.5 (16–73.5)	137.5 (59.75–325)	0.0608
Median viral load in copies/ml on admission (IQR)	345,000 (91,427–607,400)	262,450 (61,291–587,150)	391,200 (195,400–790,400)	0.8291
PJP as first AIDS-defining condition	n.a.	13/14 (92.85%)	n.a.	
Final diagnosis	**–**	PJP	See note^a^	

PJP, *Pneumocystis jirovecii* pneumonia; IQR, inter quartile range; n.a., not applicable

^a^Final diagnoses of PJP-patients: bacterial pneumonia (n = 3), TB (n = 3), disseminated Cytomegalovirus infection (n = 1) and *Mycobacterium avium* complex infection (n = 1) with lung involvement, non-Hodgkin’s T cell lymphoma (n = 1) and adenocarcinoma (n = 1) of the lung

## Results

### Patient characteristics

The records of 273 HIV-positive patients admitted during the study period were reviewed. 81 patients (29.6%) were diagnosed with ALI. Complete documentation was available for 24 patients, 18 males, and 6 females, of which 14 (58.3%) had microbiologically confirmed PJP (PJP+) and 10 (41.7%) were diagnosed with other conditions (PJP−). Clinical and demographic data are summarized in Table 2.

**Table 2:** Diagnostic performances of the lung ultrasound findings

Lung ultrasound sign	PJP+ *n* (%)	PJP− *n* (%)	*p*-value	Se (95% CI)	Sp (95% CI)	PPV (95% CI)	NPV (95% CI)	LR+ (95% CI)	LR− (95% CI)
B-lines (> 2 per view) no B-lines in the scan	14 (100%)	6 (60%)	0.0198	100% (76.84–100)	45.45% (16.75–76.62)	70% (57.64–80.01)	100% –	1.83 (1.07–3.14)	0.00 –
Pleural effusion	0 (0%)^a^	5 (50%)	0.0059	0.00% (0.00–23.16)	50.00% (18.71–81.29)	0.00% –	26.32% (16.12–39.90)	0.00 –	2.00 (1.08–3.27)
Pneumothorax	4 (28.5%)	1 (10%)	0.3577	40.00% (12.16–73.76)	90.00% (55.50–99.75)	80.00% (34.93–96.75)	60.00% (46.48–72.15)	4.00 (0.54–29.81)	0.67 (0.39–1.15)
Subpleural consolidation	14 (100%)	4 (40%)	0.0016	100% (76.84–100)	60.00% (16.24–87.84)	77.78% (62.10–88.20)	100% –	2.50 (1.17–5.34)	0.00 –
Lung consolidation, any lung consolidations with “cystic” changes at the pulmonary basis	8 (57.1%)	5 (50%)	1.0000	57.14% (28.86–82.34)	50.00% (18.71–81.29)	61.54% (42.60–77.52)	45.45% (25.95–66.46)	1.14 (0.53–2.46)	0.86 (0.36–2.04)
Lung consolidation with air bronchogram	0 (0%)	4 (40%)	0.0198	0.00% (0.00–23.16)	60.00% (26.24–87.84)	0.00% –	30.00% (20.53–41.55)	0.00 –	1.67 (1.00–2.76)
Lung consolidation with cystic pattern	8 (57.1%)	0 (0%)	0.0019	57.14% (28.86–82.34)	100% (69.15–100)	100% –	62.50 (47.65–75.32)	–	0.43 0.23–0.78

PJP, *Pneumocystis jirovecii* pneumonia; Se, sensitivity; Sp, specificity; PPV, positive predictive value; NPV, negative predictive value; LR+, positive likelihood ratio; LR−, negative likelihood ratio; CI, confidence interval

^a^One patient developed bilateral pleural effusion later during the stay in the Intensive Care Unit

The frequency of lung ultrasound findings is summarized in Table 3. B-lines, subpleural consolidations, and cystic changes were more frequently seen in patients with PJP, and reached statistical significance. In particular, B-lines and subpleural consolidations were present in all PJP+ patients in our cohort giving a sensitivity of 100%. The specificity of these two findings was low (45 and 60%, respectively). The presence of consolidations with cystic changes had a very high specificity for PJP. Pleural effusions and consolidations with linear air bronchograms were not seen in PJP+ patients.

**Table 3:** Description of lung ultrasound findings

Findings	Image	Description	Interpretation
A-lines		Horizontal lines separated from the pleural line by regular intervals that are equal to the distance between skin and pleural line Pleural line remains intact	Normal
B-lines		Vertical, hyperechoic reverberation artifacts originating from the pleural line and extending throughout the field of view Pleural line remains intact	Up to 2 B-lines per view are normal, otherwise fluid in the interstitial space is suspected
Pleural effusion		Anechoic areas that separate lung tissue from pleura and diaphragm, may be simple “black” fluid or contain complex septations Separation of visceral and parietal pleura by fluid	Collection of pleural transudate or exudate
Pneumothorax		Absence of dynamic pleural sliding, a lung point represents the transition between loss of pleural sliding and the return of pleural sliding (the border of the pneumothorax) Separation of visceral and parietal pleura by air	Air in the pleural space
Subpleuric consolidation		Small hypoechoic areas (< 2 cm) disrupt the visceral pleura Parietal pleura acquires a “ragged” or “shredded” appearance	Small consolidations are present on the visceral pleura itself
Consolidation with linear bronchogram		Larger irregular hypoechoic areas containing linear hyperechoic air bronchograms (bright areas with linear arrangement casting a vertical shadow artifact)	Alveolar parenchymal involvement, with air trapped in the obstructed airways
Consolidation with “cystic bronchogram”		Larger irregular hypoechoic areas disrupting the visceral pleura with small disseminated hyperechoic areas within consolidated lung (without marked shadow artifacts)	Alveolar parenchymal involvement, possible cystic changes as explanation for the echogenic areas

As most HIV-positive patients admitted for acute lung disease underwent routine CXR and CT scan, we were able to compare the POCUS findings with radiologic findings from other modalities. Examples from three patients are shown in Table 4. We found a strong correlation between the imaging pattern seen on ultrasound and that of other imaging modalities. Of the six PJP+ patients without “cystic changes,” three had CT scans demonstrating normal air-filled lung throughout the subpleural region, two had only intralobular opacities, and one patient had physical limitations to the scan (pectus excavatum).

**Table 4:** Comparison of findings on CT scan, CXR, and ultrasound of three patients with *Pneumocystis jirovecii* pneumonia PJP

	CT scan	CXR	Ultrasound
Patient 1			
Patient 2			
Patient 3			

The infection spares the subpleural areas in patient 1, involves mainly the subpleural areas in patient 2, and involves wider lung parenchyma in patient 3

## Discussion

This is to our knowledge the first study systematically reporting POCUS findings of the lung in HIV-related PJP. Ultrasound patterns for several lung diseases in HIV-positive patients have been described in a recently published case series [7]; however, the diagnostic accuracy of chest POCUS in this patient population has never been assessed. Our study suggests that there are thoracic ultrasound findings with significant predictive value for PJP in HIV-positive patients. B-lines, subpleural consolidations and cystic changes are suggestive of PJP. Lung consolidation with air bronchograms and pleural effusion should prompt suspicion of other etiologies.

In particular, the finding of “cystic changes” needs to be highlighted, as it was very specific in our cohort. The sign was characterized by a hypoechoic consolidation including widely scattered echogenic regions suggestive of air-containing cysts. The pattern was consistently present in serial scans of patients and seems to correlate with the type of lung consolidation seen on CT scan. The typical CT features include a “ground glass” pattern, septal thickening, and walled cysts next to the areas of ground-glass attenuation [14]. These may represent pathological changes of lung tissue with filling of the bronchioles and alveoli with foamy debris and macrophages as well as fungal cysts demonstrated in autopsies [18].

Our results should be considered preliminary, given the small numbers and the fact that a single operator performed the ultrasounds. The strength of this study is the use of a referral setting where all diagnostic methods considered to be the gold standard for diagnosing PJP (bronchoscopy, PCR, and CT scan) are routinely available. The discharge diagnosis in our population carries thus a high level of certainty.

Consolidation with “cystic changes,” the most specific ultrasound finding, was only detected in 57% of PJP+ patients and thus has a suboptimal sensitivity. This may be in part due to the inherent limitations in scanning critically ill, dyspneic patients. Also the inhomogeneous spatial distribution of the inflammatory changes may add to this problem. Early stages of the infection seem to spare the subpleural areas [19]. Normal air-filled lung prevents ultrasound from detecting underlying areas of consolidation. Nevertheless, the presence of the pattern seems helpful to “rule-in” the diagnosis.

While no single finding yielded optimal sensitivity and specificity a combination of the described findings on thoracic ultrasound could be helpful in informing clinicians about the likelihood of various lung pathologies in HIV+ patients admitted with ALI.

## Conclusion

POCUS is useful in diagnostic work-up of HIV-positive patients with acute lung disease and ultrasound patterns can help in diagnosing PJP. The use of lung POCUS poses an attractive option for HIV-patients as it combines affordability, non-invasiveness, and availability at patients’ bedside regardless of clinical condition. This is even more relevant in resource-limited settings where the majority of patients reside. Our case series should encourage larger prospective studies to validate the described thoracic ultrasound findings in HIV-positive patients.

## Additional files

**Additional file 1: Clip SA.** Subpleural consolidation: a hypoechoic area is visible in the superficial parts of the lung less than 2 cm below the pleural line.

**Additional file 2: Clip SB.** Lung consolidation with linear air bronchogram: large hypoechoic area (consolidation) visible containing hyperechoic bright areas casting a vertical shadow artifact. The bright areas are distributed in a linear pattern representing air trapped in the bronchi (sonographic air bronchogram).

**Additional file 3: Clip SC.** Lung consolidation with cystic changes: large hypoechoic area (consolidation) disrupting the visceral pleura with small, diffusely disseminated hyperechoic areas within the consolidated lung (without marked shadow artifacts). The spatial distribution of is not linear as in clip B.

## Abbreviations

CXR: X-ray
POCUS: point-of-care ultrasound
TB: tuberculosis
PJP: *Pneumocystis jirovecii* pneumonia
CT: computed tomography
ART: antiretroviral therapy
BAL: bronchoalveolar lavage
ALI: acute lung injury
